# Implementation of healthcare guidelines in public health: an umbrella review

**DOI:** 10.1186/s12889-026-28065-x

**Published:** 2026-06-19

**Authors:** Hristiyanna I. Ivanova, Jacqueline A. Deurloo, Caren I. Lanting

**Affiliations:** Expertise Group Child Health, the Netherlands Organization for Applied Scientific Research (TNO), PO Box 2215, Leiden, 2301CE the Netherlands

**Keywords:** Umbrella review, Healthcare guidelines, Public health, Implementation, Barriers and facilitators, Implementation strategies

## Abstract

**Background:**

Healthcare guidelines are intended to standardize health practitioners’ practice and improve patient outcomes, yet their implementation in public health is often inconsistent. This umbrella review aimed to identify barriers and facilitators influencing guideline implementation and to provide an overview of effective strategies to enhance guideline adherence in public health.

**Methods:**

Systematic reviews and meta-analyses, published between 2014 and 2024, were identified through searches in PubMed (Q4 2024) and Scopus, CINAHL, and the Cochrane Database (Q2 2026). After applying the inclusion criteria, these were assessed for methodological quality using the AMSTAR-2 tool. Barriers, facilitators, and strategies were categorized using the Measurement Instrument for Determinants of Innovations (MIDI) framework to provide a comprehensive overview.

**Results:**

Thirteen systematic reviews were included, most of which conducted qualitative syntheses, with only one meta-analysis identified. Organizational determinants were the most frequently reported barriers, including limited resources, insufficient coordination, and lack of management support. Factors related to healthcare professionals, such as perceived relevance of guidelines and alignment with practice, also influenced implementation. Facilitators included perceived usefulness of guidelines, professional engagement, and social support.

A wide range of implementation strategies was identified, targeting organizational structures, end-users, and guideline characteristics. Educational approaches were the most applied but showed inconsistent effectiveness, especially when used in isolation. Evidence on the added value of multi-component strategies was mixed, with no single approach consistently associated with successful implementation.

**Conclusion:**

Effective implementation of healthcare guidelines in public health requires a comprehensive approach addressing context-specific determinants. Although implementation challenges are shared across healthcare, the structural characteristics of public health often amplify organizational and end-user barriers. No single strategy ensures consistent success; instead, effectiveness depends on tailoring implementation efforts to specific settings and determinants. Further high-quality research is needed to identify effective implementation strategies and bridge the gap between evidence and daily public health practice.

**Registration:**

This umbrella review was not registered in a publicly accessible database prior to its conduct.

**Supplementary Information:**

The online version contains supplementary material available at https://doi.org/10.1186/s12889-026-28065-x.

## Introduction

Healthcare guidelines are recommendations based on scientific evidence, aiming to improve the process of healthcare delivery as well as patient outcomes by guiding the practice of professionals in the clinical and public health sector [1]. These guidelines are designed to standardize healthcare practices across various settings and among practitioners. By incorporating patient values, they assist healthcare professionals and patients in making evidence-informed decisions about appropriate care in specific situations [2].

Keeping up with new evidence presents a challenge for healthcare professionals, who must continually update and adapt their practices accordingly to reflect the latest evidence. To support this process, healthcare guidelines play a crucial role by synthesising, reviewing, and translating scientific evidence into actionable recommendations for practice. These guidelines are typically developed using an accepted step-by-step methodology, such as GRADE (Grading of Recommendations Assessment, Development, and Evaluation) to ensure their consistency and high quality [3, 4].

The effectiveness of guidelines in improving healthcare practice and patient outcomes depends strongly on their implementation [1]. Implementation is the process of planned, deliberate activities aimed at putting well-defined actions and programs into practice [5, 6]. In this context, it refers to the adherence of healthcare professionals to guidelines in their practice.

However, discrepancies in the uptake and use of guidelines are observed in clinical and public healthcare practices worldwide [7, 8]. For instance, Dutch studies report significant variation in adherence to guidelines for general practitioners (52% − 95%) and to discipline-specific public health guidelines (14% − 94%), such as those used in Youth Health Care (YHC) [9, 10]. Fixsen et al. [5] emphasize that, despite the potential effectiveness of interventions (i.e., healthcare guidelines), their ineffective implementation often leads to suboptimal use in practice and thus compromised patient outcomes.

This signifies the need for a better dissemination of evidence-based guidelines, which can be achieved through well-designed implementation strategies [8, 11, 12]. These strategies encompass various methods to enhance guideline adoption and should be a fundamental component in any guideline development plan.

While much literature exists on the implementation of guidelines in the clinical setting [13–19], these findings may not translate directly to the public health sector, which presents distinct challenges due to its population-level focus, multi-sectoral involvement, emphasis on prevention, equity of care, and broader societal goals.

Despite its importance, there is a significant knowledge gap regarding how guidelines are adopted and applied in public health practice. This highlights the need for an updated, comprehensive review to better understand the implementation dynamics within this field. An umbrella review is particularly suitable for this purpose, as it synthesizes and evaluates findings from existing systematic reviews, offering the highest level of evidence [20].

This umbrella review aims to address this knowledge gap by assessing the adherence to guidelines in the public health sector. Specifically, it seeks to: (1) identify the barriers and facilitators impacting their intended use, and (2) provide an overview of implementation strategies improving adherence outcomes. The insights from this review will serve to inform future guideline development and ultimately contribute to better patient outcomes.

## Methods

### Search strategy and eligibility criteria

This review was conducted following the Preferred Reporting Items for Overviews of Reviews (PRIOR) methodology [21] (see Supplementary file 1). Systematic reviews and meta-analyses, employing a structured and transparent methodology to identify, select, critically appraise and synthesize findings from multiple studies, were identified via PubMed. The search took place in Q4 of 2024 and employed the following key terms:

1. (“Diffusion of Innovation“[Mesh] OR implement*[Title] OR adoption[Title] OR quality improvement[Title] OR disseminat*[Title] OR diffusion[Title] OR knowledge transfer[Title])

*AND*

2. (“empirically supported treatment“[Title] OR “evidence-based practice“[Title] OR “evidence-based treatment“[Title] OR “evidence-based intervention“[Title] OR “guideline“[All Fields] OR “guidelines as topic“[MeSH Terms])

*AND*

3. (strateg*[Title] OR intervention*[Title] OR program*[Title] OR plan*[Title] OR process*[Title] OR model*[Title] OR framework*[Title] OR barriers[Title] OR limitations[Title] OR facilitators[Title] OR facilitating factors[Title])

In Q2 of 2026, we conducted a second literature search in Scopus, CINAHL and the Cochrane Database of Systematic Reviews, using comparable search terms to ensure comprehensive coverage of the multidisciplinary nature of implementation research in public health. Complete search strategies for all databases are available in Supplementary file 2.

Retrieved articles were considered for inclusion if they (a) were systematic reviews or meta-analyses written in English or Dutch, (b) were published between Q1 2014 and Q4 2024 in a peer-reviewed journal, (c) assessed barriers and facilitators to guideline adherence and/or strategies/theories of behaviour change aimed at improving guideline implementation, and (d) were conducted in public healthcare settings, specifically focusing on public health guidelines. Reviews focusing exclusively on clinical care without relevance to public health guideline implementation were excluded. However, given the limited availability of reviews conducted solely in public health settings, reviews including a combination of public health and clinical care settings were considered eligible when they addressed guideline implementation in preventive or population-oriented contexts.

In the Netherlands, for example, public health is delivered through organizations such as Youth Health Care (YHC) and Municipal Public Health Service (GGD), which focus on preventive care and health promotion at the population level. However, internationally, the structure and delivery of public health services vary considerably, depending on national structures, resources, and priorities. Therefore, for the purposes of this umbrella review, studies conducted in primary care, community centers, school health services, and similar settings were considered. This approach acknowledges the diversity of public health delivery models worldwide and ensures that the review captures relevant evidence across different healthcare systems.

Unpublished articles, case-reports, conference abstracts, and non-peer-reviewed sources were excluded. The main outcomes of interest regarding implementation strategies were (1) measures of professional practice effectiveness (such as guideline adherence, change in professional practice, documentation compliance, and referral patterns), (2) improved patient outcomes and (3) cost-effectiveness.

### Data extraction and quality assessment

Screening and selection of literature occurred in two stages: initial screening of titles and abstracts with the use of ASReview software [22], followed by a manual full-text screening. Similarly to previous reviews [23, 24], 100 random titles and abstracts were screened in ASReview to estimate the total number of potentially relevant studies and to initialise the active learning model. Based on this initial sample, an upper 95% confidence limit of the number of relevant studies was calculated to guide the stopping point. In line with the SAFE (Stopping Active learning in sEarch) procedure [25], multiple stopping criteria were combined to ensure a balance between efficiency and sensitivity. Screening was continued until the estimated upper bound of relevant studies had been identified and a sequence of 100 consecutive records was labeled as irrelevant. To validate the stopping decision and minimize the risk of missing relevant studies, a validation step was performed by screening 50 of the lowest ranked (i.e., least relevant) records as predicted by the model. The absence of relevant studies within this subset was taken as an indication that no further relevant studies remained in the dataset.

Any uncertainties or disagreements were resolved through discussion with all authors. The following data were extracted: (1) study characteristics, (2) study design and healthcare setting, (3) objectives, (4) outcome measures, (5) number of included studies and (6) quality appraisal. Additionally, findings were systematically summarized in a structured overview across reviews.

The methodological quality of the literature was appraised using AMSTAR-2 (A MeaSurement Tool to Assess Systematic Reviews) [26]. Each review was evaluated based on the 16 items of the tool, and an overall rating was assigned: high (no or one noncritical flaw), moderate, low or critically low quality (more than one critical flaw with or without noncritical flaws).

### Data analysis

We assessed overlap of primary studies across included systematic reviews by constructing a citation matrix (primary study × systematic review) and calculating the Corrected Covered Area (CCA) as described by Hennessy et al. [27]. Reviews without an extractable list of primary studies (e.g., [28]). were excluded from the CCA calculation but included in the qualitative synthesis.

The Measurement Instrument for Determinants of Innovations (MIDI) tool was used to analyze the evidence on barriers, facilitators and strategies of guideline implementation [29]. This validated tool is specifically designed to categorize key determinants that can influence the successful implementation of innovations. In this review, the healthcare guidelines were considered the innovation to be implemented. The MIDI tool divides determinants into four domains: those related to the innovation itself (e.g., procedural clarity, complexity, relevance to the patient); those related to the end-user (i.e., person adopting the innovation), such as perceived benefits/drawbacks, professional obligation or sufficient knowledge; those regarding the organization (e.g., staff capacity and financial resources); and those regarding the socio-political context (e.g., legislation and regulations). To provide a comprehensive overview of the identified barriers and facilitators to guideline implementation, a template was developed. This template enabled thematic analysis and standardized the categorization of determinants according to the elements within the MIDI domains. The same procedure was applied to the implementation strategies. However, because some strategies address multiple elements within a single domain, a decision was made to categorize them only at the domain level. Disagreements were discussed with all authors until consensus was reached.

To assess the impact of methodological quality, the results were further stratified by their overall AMSTAR-2 rating. This allowed for comparison of the outcomes across reviews and facilitated a sensitivity analysis, helping to identify potential discrepancies and strengthen the interpretation of the evidence.

## Results

### Summary of included reviews

**Fig. 1:**
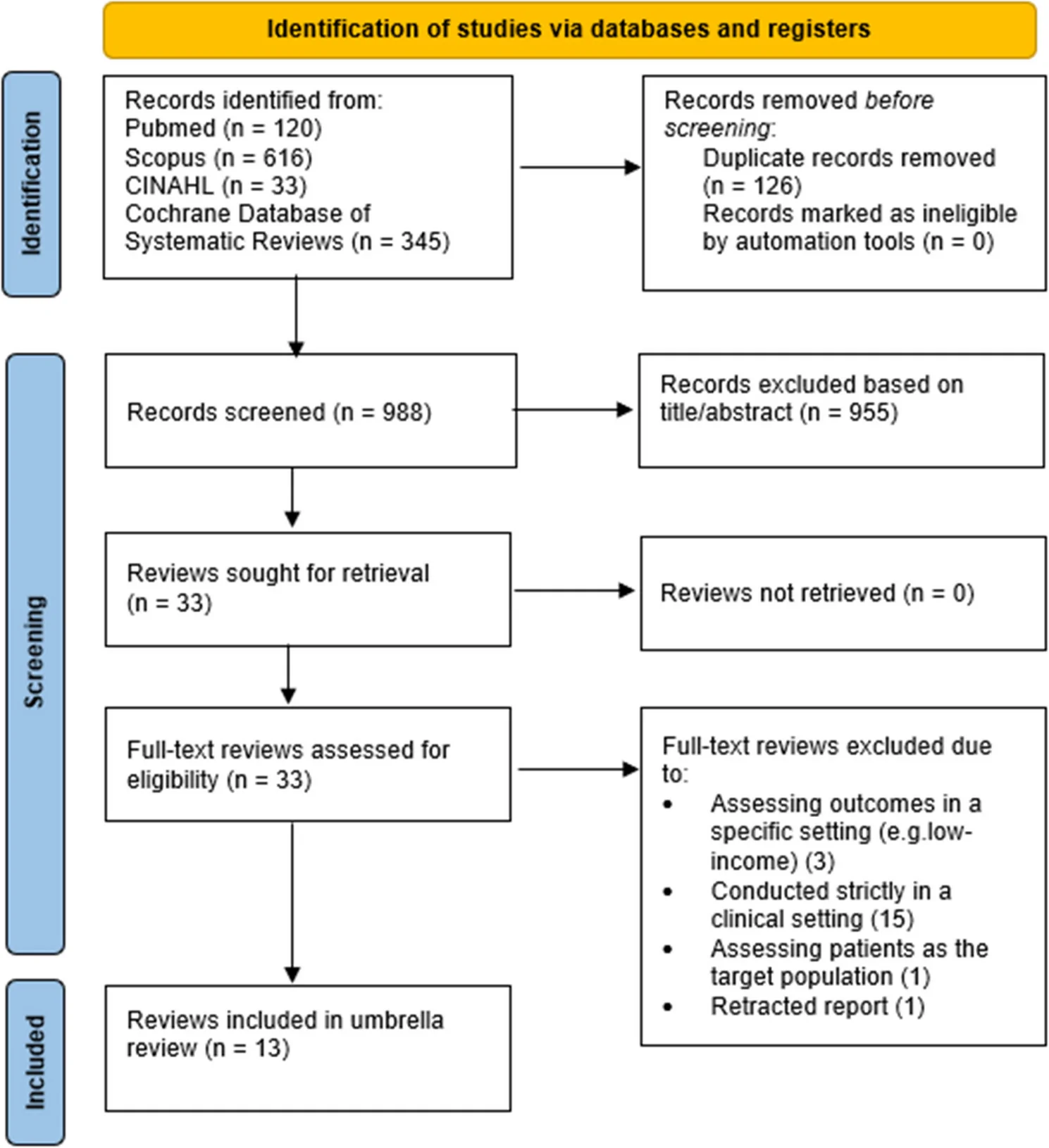
PRISMA flow diagram of search results [43]. A full list of excluded records is available in Supplementary file 3

A total of 1114 articles were identified (Fig. 1). After deduplication and title and abstract screening, 33 full-text reviews were retrieved, and 13 met the preset inclusion criteria (see Table 1). Across the 12 systematic reviews with extractable individual studies, the citation matrix contained 17 primary studies appearing in more than one review and no primary study appearing in more than two reviews (maximum overlap = 2). The overall CCA was 0.54%, indicating minimal overlap according to established thresholds [30]. In line with current methodological guidance, this low level of overlap was interpreted as negligible, and no adjustments for overlap were deemed necessary.

**Table 1:** Overview of included systematic reviews and meta-analyses

Reference	First author	Year	Study design and healthcare setting	Objective(s)	Number of included studies	Outcome measures	Quality appraisal (AMSTAR-2)
[31]	Kovacs	2018	Systematic review and meta-analysis Primary healthcare	Evaluate the effect of intervention methods on the guideline adherence of primary care providers (PCPs).	36	Change in professional practice, impact on patient outcomes	Low
[32]	Heslehurst	2014	Systematic review Public health midwifery	Identify the determinants of healthcare professionals’ practice in relation to maternal obesity and weight management.	25	Implementation of pregnancy weight management and obesity guidelines by healthcare professionals	Critically low
[33]	Cassidy	2021	Systematic review Nursing care	Describe the use and effects of implementation strategies to facilitate the uptake of guidelines focused on nursing care.	41	Implementation of guidelines in nursing care Change in professional knowledge, professional practice, patient health status outcomes, resource use, and expenditures	Moderate
[34]	Häggman-Laitila	2016	Systematic review Nursing care	Review the literature on the outcomes of educational interventions relevant to nurses with regard to guideline implementation.	13	Guideline implementation using educational interventions in nursing Change in patient outcomes	Critically low
[35]	Mathieson	2018	Systematic mixed-studies review Community nursing	Identify the strategies required for, and the barriers and facilitators to, successful implementation	22	Implementation of evidence-based practice in community nursing	Critically low
[36]	Spoon	2020	Systematic review Nursing care	Provide an overview of strategies used to implement nursing guidelines in all nursing fields, as well as the effects of these strategies on patient-related nursing outcomes and guideline adherence	54	Implementation of nursing guidelines and impact on patient-related nursing outcomes	Low
[37]	Kovacs	2019	Systematic review Primary care	Review the economic evaluation of guideline implementation in primary care	39	Economic outcomes related to process of care and patient outcomes (e.g., quality of life)	Low
[38]	Mergelsberg	2023	Systematic review Primary care	Assess active ingredients, mechanisms of action, and implementation fidelity of interventions aimed at improving smoking cessation care	15	Impact on guideline adherence, counselling behaviour and quality of care	Critically low
[39]	Duda-Sikuła	2023	Systematic review Primary care and healthcare systems addressing health prevention	Identify best practice guidelines and policies influencing prevention strategies for chronic disease patients and to examine barriers and facilitators for their implementation	47	Implementation outcomes (implementation of prevention strategies and guidelines); system-level, organizational, and individual-level determinants	Critically low
[40]	Ray	2022	Systematic review Primary care	Synthesize evidence on primary care providers’ practices and perceived barriers and facilitators of implementing guideline-recommended childhood obesity prevention practices	50	Implementation-related outcomes (practice implementation, behavioural determinants, barriers/facilitators)	Moderate
[28]	Leeman	2015	Systematic review Community-based and public health settings (non-clinical prevention interventions)	Identify capacity-building strategies used to support practitioners in implementing evidence-based prevention interventions and assess their effectiveness	29	Implementation-related outcomes (adoption, implementation, practitioner capacity, planning behaviours)	Low
[41]	Agarwal	2021	Cochrane review Primary healthcare	Assess the effects of digital clinical decision-support systems (CDSS) delivered via mobile devices on quality of care and adherence to guidelines in primary care	8	Change in professional practice (guideline/protocol adherence), patient health behaviour, patient health outcomes, patient satisfaction	High
[42]	Jensen	2016	Systematic review and meta-analysis Primary healthcare	Identify and assess the cost-effectiveness of strategies for implementing low back pain guidelines in primary care and evaluate transferability of results	3	Change in professional practice (e.g., referrals), patient outcomes (e.g., functional capability, quality of life), cost-effectiveness	Critically low

All reviews were published from 2014 to 2023 and conducted a qualitative synthesis of the evidence. Only one review performed a meta-analysis to quantitatively combine findings [31]. The included reviews synthesized evidence from a wide range of primary study designs, such as RCTs, cluster-randomized trials, controlled before–after studies, cohort studies, and other quasi-experimental designs, as well as observational studies (e.g., cross-sectional surveys) and qualitative or mixed-methods studies examining healthcare professionals’ experiences and behaviours.

The number of primary studies ranged from 3 to 54, with an average of 29 studies per review. The reviews covered a range of healthcare fields, including primary healthcare practice (*n* = 7) [31, 37–42], (community) nursing care (*n* = 4) [33–36], public health midwifery (*n* = 1) [32] and community-based public health [28]. Across reviews, evidence was drawn from both public health settings (e.g., community nursing or preventive services) and clinical care settings (e.g., outpatient care) [28, 33–36, 38, 40, 41], or combined findings from public health guidelines (focused on population-level prevention) with those addressing clinical guidelines (focused on individual patient care) [31, 32, 37, 39, 42].

Two reviews focused exclusively on barriers and facilitators of guideline adherence [32, 40], while six examined strategies to enhance guideline implementation [28, 31, 33, 34, 38, 41]. Three reviews examined both determinants of guideline adherence and implementation strategies [35, 36, 39]. Two reviews primarily assessed the cost-effectiveness of guideline implementation [37, 42]. An overview of the identified barriers and facilitators, categorized according to the MIDI instrument, alongside the implementation strategies reported in the included reviews, is presented in Table 2.

**Table 2:** Overview of barriers and facilitators (based on the MIDI instrument [29]) and implementation strategies

Barriers to guideline implementation related to the:
End-user (i.e., the adopting healthcare professional)
Lack of sufficient knowledge and skills [30, 33, 37, 38]
Incompatible to professional obligations [33, 38]
Low self-efficacy [38]
Low outcome expectations [38]
Limited client cooperation [30, 38]
Organization
Unsettled organization [33]
Lack of coordination [33] Lack of formal ratification by management [34, 37 , 38]
Lack of available time [30, 33, 37, 38]
Lack of replacement when staff leave [38]
Insufficient staff capacity [33, 37]
Limited/ inadequate financial resources [33, 37, 38]
Limited/ inadequate material resources and facilities [37,38]
Facilitators to guideline implementation related to the:
Innovation
Complexity [33] Observability [33] Compatibility [33, 37]
Correctness [37] Relevance for client [33, 37]
End-user (i.e., the adopting healthcare professional)
Personal benefits [33]
Client cooperation [30, 38]
Outcome expectations [30, 38]
Social support [30, 33]
Subjective norm [30, 33]
Single-component implementation strategies related to the:
Innovation
Adaptation of guidelines to local context [31, 33, 34]
Participatory/collaborative approaches during guideline development [31]
Guideline (implementation) support tool [36, 39, 40]
End-user (i.e., the adopting healthcare professional)
Educational strategies [29, 31, 33, 34, 36, 37, 39]
Educational outreach visits [29, 31, 34, 37]
Ongoing support following initial training [31, 33, 39]
Incentives [39]
Organization
Implementation teams [37]
Local opinion leaders [31, 33, 34]
Organizational policy changes [31, 36, 37, 39, 40]
Leadership/ stakeholder involvement during implementation [36, 40]
Audit and feedback [31, 34]
Automated reminders [29, 31]
Resource allocation [33]
Multi-component implementation strategies
Educational strategies with external facilitation and local adaptation of the guideline [31, 33]
Educational strategies with organizational policy change [31, 33]
Educational strategies with audit and feedback, and opinion leaders [29, 31, 34]
Educational strategies with support tools [29, 32, 40]

### Quality of included reviews

The quality of the included reviews varied: the majority were rated as critically low [32, 34, 35, 38, 39, 42] and only one was of high quality [41], while the remaining reviews were of low to moderate quality (see Table 1 for the full quality assessment). Reviews rated as critically low showed notable methodological weaknesses, such as incomplete search strategies, limited or non-standard risk of bias assessment, insufficient consideration of heterogeneity and lack of transparency regarding excluded studies.

### Barriers to guideline implementation

Five reviews discussed barrier assessment [32, 35, 36, 39, 40]. Of the identified barriers, 42% were related to the end-user (i.e., the adopting healthcare professional), and 58% to the organization where the guidelines were implemented. No determinants regarding the innovation itself or socio-political context were reported.

### Barriers related to the end-user

“Knowledge” was identified as a barrier by four reviews [32, 35, 39, 40]. The lack of sufficient knowledge about recommended practices among healthcare professionals, as well as the absence of formal training on the use of healthcare guidelines, hindered their effective implementation. In addition, a lack of academic and communication skills also emerged as a barrier, particularly in counseling [32, 35, 40]. This included difficulties in addressing sensitive topics with patients, such as personal discomfort, anxiety or embarrassment when discussing these issues, fear of offending patients or provoking negative reactions or stigma, and concerns about damaging the practitioner–patient relationship.

Other limitations within this domain included “professional obligation” [35, 39, 40],“self-efficacy” [40], “outcome expectations” [40] and “client cooperation” [32, 40]. Healthcare practitioners who perceive guidelines as incompatible with their roles or values, do not see guideline adoption as part of their professional responsibilities or disagree with its contents, or are skeptical about their effectiveness, are less likely to incorporate guidelines into their practice [35, 39, 40]. This also applies to practitioners who lack the confidence that they are able to implement change (i.e., low self-efficacy) [40] or practitioners who perceive patients as lacking motivation or engagement [32, 40].

### Barriers related to the organization

Various organizational characteristics also appeared to influence the extent of guideline implementation.

Mathieson et al. reported on the MIDI elements ‘unsettled organization’ and ‘coordination’ [35]. Infrastructural changes, such as restructuring of services or teams in an organization, require time and are prioritized in the daily practice of healthcare professionals, reducing the attention and effort they can devote to guideline implementation. Additionally, the absence of an appointed group of professionals, responsible for coordinating and monitoring the use of healthcare guidelines within an organization, hinders effective guideline implementation in practice.

Other organization-specific barriers were related to the lack of “formal ratification by management” [35, 36, 39, 40], “time available” [32, 35, 39, 40], “replacement when staff leaves” [40], “staff capacity” [35, 39], “financial resources” [35, 39, 40] and “material resources and facilities” [39, 40]. A lack of leadership support and motivation towards end-users, along with the failure to establish formal arrangements regarding guideline use within the organization, limited their effective uptake [36, 39, 40]. Insufficient continuity of care, often caused by staff turnover, hindered the development of stable practitioner–patient relationships, thereby negatively affecting guideline implementation [40]. Staff shortages and insufficient personnel further restricted implementation by limiting the capacity to meet service demands [35, 39].

Financial and time constraints also impeded implementation, as limited funding reduced access to protocols, training opportunities, and support structures, while limited consultation time, and competing priorities restricted the application of guidelines [32, 35, 39, 40]. Finally, a lack of material resources, such as equipment, decision-support tools, and patient educational materials, as well as restricted access to referral options, limited the practical application of guideline recommendations [39, 40].

### Facilitators to guideline implementation

Four systematic reviews explored factors supporting the implementation of guidelines [32, 35, 39, 40]. Half of these factors were associated with the innovation itself and half concerned the end-user.

### Facilitators related to the innovation

Within this domain of the MIDI tool, the following were identified as key elements supporting the adherence of healthcare practitioners to guidelines - “complexity” [35], “observability” [35], “compatibility” [35, 39], “correctness” [39] and “relevance for client” [35, 39]. Practical aspects of the guidelines themselves, such as ease of use, cost-effectiveness and time-efficiency, played a significant role in their acceptance. Higher uptake was observed when guidelines were perceived as compatible with existing professional values and routines, and when they could be adapted to the local, social, and cultural context [35, 39]. In addition, their evidence-based nature, enhanced credibility [39], while visible beneficial outcomes, such as improved patient care or stronger practitioner-patient relations, further supported adoption by professionals [35, 39]. Mathieson et al. emphasized that involving healthcare practitioners early in the guideline development process fosters a sense of ownership and increases the perceived relevance and applicability of guidelines in practice [35].

### Facilitators related to the end-user

Facilitators identified within this domain included “personal benefits” [35], “patient cooperation” [32, 40], “outcome expectations” [32, 40], “social support” [32, 35] and “subjective norm” [32, 35]. Healthcare professionals were more inclined to adopt guidelines when their use contributed to personal benefits, such as professional development or gaining new knowledge or developing new skills [35]. Positive outcome expectations also played a key role: practitioners who believed that their preventive actions would lead to meaningful improvements in patient outcomes were more motivated to implement guidelines [32, 40]. Furthermore, the support, cooperation, and feedback from both patients and colleagues were seen as influential in shaping practitioners’ practice, thereby facilitating guideline implementation [32, 35]. Even when practitioners questioned their own readiness or competence to implement guidelines, strong and trust-based pre-existing professional-patient relations appeared to encourage successful adoption.

### Implementation strategies

Ten systematic reviews investigated implementation strategies aiming to enhance guideline adoption in healthcare practice [28–42]. Each review assessed single-component implementation strategies, while five also examined strategies that combined multiple components (i.e., multi-component strategies) [31, 33, 34, 36, 41].

### Single-component strategies

Half of the single-component strategies focused on addressing organizational challenges. Notably, none of the strategies targeted issues within the socio-political context.

#### Implementation strategies related to the innovation

Three implementation strategies related to the innovation were identified – adapting guidelines to the local context [33, 35, 36], using participatory/collaborative approaches [33] and the provision of practical tools to support guideline use in practice [28, 38, 41]. Contextual adaptation of the guidelines includes tailoring evidence-based recommendations to fit the specific practices and conditions of a local setting. This approach builds on the local knowledge and practice, making it easier for healthcare practitioners to integrate guidelines into their work. Participatory/collaborative strategies focus on the involvement of key stakeholders (e.g., healthcare professionals, patients, etc.) throughout all stages of the guideline development process. Considering their needs, insights and priorities leads to the development of healthcare guidelines, which are relevant and widely accepted.

Additionally, providing practical tools, such as simplified guideline versions, physical information cards, implementation protocols or accessible web portals, supports implementation by facilitating day-to-day guideline use and improving accessibility for healthcare professionals in practice [28, 38]. In contrast, more advanced tools, such as digital clinical decision-support systems (CDSS), provide active, real-time support by guiding healthcare professionals through evidence-based decision-making processes, thereby directly supporting the application of guidelines during clinical encounters [41].

#### Implementation strategies related to the end-user

Within this domain, educational strategies were the most frequently used approaches to enhance the implementation of healthcare guidelines and were reported across all reviews reporting on implementation strategies [28, 31, 33, 35, 36, 38, 39]. While the approach varied, most of the strategies involved pre-planned educational sessions for healthcare practitioners prior to guideline rollout. Formats varied widely, including face-to-face lectures, workshops, seminars, web-based sessions, group discussions, and video presentations. Both passive (e.g., information provision) and active learning approaches (e.g., small group work, role-play, case-based learning, and patient simulations) were used to engage healthcare professionals.

In some instances, external facilitators conducted outreach visits to deliver training, typically in a one-way format aiming to provide expert guidance [31, 33, 36, 39]. Inter-professional education was also applied in several cases, promoting collaboration and mutual learning across disciplines and thereby supporting a more integrated approach to guideline implementation. Moreover, passive educational tools, such as distributing a print of the guidelines, supplementary materials or self-study e-learning modules were also reported.

Another strategy targeting end-users involved providing ongoing support following initial training [28, 33, 35]. This support could be provided from other staff members, creating opportunities to exchange positive and negative experiences with the use of the guidelines, or from the guideline developers through direct contact or via phone. Moreover, support from management also played a vital role by reinforcing expectations, allocating resources, and cultivating a supportive work environment that encourages the use of evidence-based practices.

In addition, incentives were reported as a strategy to motivate engagement in implementation activities, such as offering financial compensation or training opportunities (e.g., scholarships), thereby encouraging healthcare practitioners to participate in and sustain guideline-related practices [28].

#### Implementation strategies related to the organization

Seven distinct strategies related to the organization were identified.

A commonly used approach involves assigning specific roles to facilitate the implementation process. This included the formation of dedicated implementation teams, designating key individuals to lead implementation efforts, and appointing staff to monitor the progress [39]. In some cases, external experts were brought in to provide support and guidance throughout the implementation phase [33]. Another strategy included leveraging the influence of knowledgeable staff members as local opinion leaders, whose expertise and peer credibility promoted the adoption of guidelines within teams [33, 35, 36].

Organizational policy changes as a way to enhance guideline use were also reported [28, 33, 38, 39, 41]. This strategy involved reforming current practice and integrating the guidelines within formal protocols, or standard operating procedures, therefore ensuring that their use becomes a required part of routine care. In addition, broader governance approaches, such as stakeholder engagement and leadership involvement, were reported to support implementation at a systems level [38, 41].

Another strategy used to encourage changes in current practice and improve guideline adherence is ‘audit and feedback’ [33, 36]. This involves collecting data on guideline use, either using health records or direct observations by a third party. These findings are then summarized and shared with healthcare professionals to encourage reflection and promote improvements in practice.

Another organizational component included the use of automated reminders containing the guideline essentials [31, 33]. These reminders were delivered via monthly email reminders or a pop-up window, integrated into health records, serving as a clinical decision support tool.

Mathieson et al. briefly reported on organizational support, particularly through allocating resources to boost implementation of evidence-based healthcare guidelines [35]. This typically involves providing healthcare professionals with the necessary time to become familiar with new practices and investing resources to stimulate the integration of guidelines into standard care.

#### Multi-component strategies

The reviews also highlighted various combinations of implementation approaches to support the adoption of guidelines: multi-component strategies. These strategies are often centered around educational interventions, supplemented by organizational, contextual, or interpersonal components.

A commonly reported combination involved pairing educational strategies with external facilitation and local adaptation of the guideline [33, 35]. Another frequently mentioned approach was the combination of educational efforts with organizational policy change [33, 35]. In these cases, guideline implementation was supported by embedding the recommendations into institutional protocols to ensure long-term sustainability.

Several reviews also described strategies that combine education, audit and feedback, and the involvement of opinion leaders [31, 33, 36]. This configuration aimed to reinforce learning and promote adherence to guidelines through performance feedback, as well as the influence of respected peers within the organization.

Lastly, education was also frequently combined with (technical) support tools, such as reminders and information cards to support consistent application of guidelines in daily practice [31, 34, 41].

### Effectiveness of implementation strategies

Seven of the thirteen reviews [28, 31, 33, 34, 36, 37, 41] examined the effectiveness of implementation. Only one review conducted a meta-analysis [31], and one assessed the cost-effectiveness of strategies [37].

Educational strategies were widely used in nearly all included studies (98%) but generally had low to moderate effectiveness [36]. While five studies showed improvements in professional practice following educational interventions, only two demonstrated an impact on professional knowledge, and two showed no measurable effect on patient health outcomes [33]. Passive educational methods (i.e., the distribution of materials) were consistently the least effective [31].

Kovacs et al. found that single-component strategies demonstrated a greater average effect size (Standardized Mean Difference (SMD) = 0.27 [95% CI 0.17, 0.38]) compared to multi-component strategies (SMD = 0.13 [95% CI 0.06, 0.19]) [31]. These approaches were particularly effective in improving healthcare practitioners’ knowledge (SMD = 0.39) and had a moderate impact on diagnostic practices (SMD = 0.22) but showed little to no effect on patient health outcomes.

In contrast, other reviews suggested that combining multiple components may enhance effectiveness. Häggman-Laitila et al. observed that combining multiple elements within a strategy yielded better outcomes, including improved practitioner confidence, sustained changes in professional practice, and improved quality of care [34]. Similarly, over two-thirds of studies employing multi-component strategies (68%) demonstrated significant improvements in patient outcomes or guideline adherence, although the median improvement did not differ significantly from single-component strategies (20.1% vs. 13.8%, *p* = 0.95) [36].

Cassidy et al. found positive outcomes in 36 studies evaluating multi-component strategies, particularly regarding resource use (80%), professional knowledge (64%), and outcomes related to financial outcomes (63%) [33]. Leeman et al. similarly found that training, technical assistance, and support tools were generally effective in increasing guideline adoption (10/12 studies) and implementation (9/10 studies), although their effects on knowledge, skills, and planning behaviors were mixed [28]. Agarwal et al. also reported mixed effectiveness of digital decision‑support tools, with uncertain effects on provider adherence and patient outcomes [41].

Economic evaluations, particularly from Jensen et al., indicated that implementation strategies (including educational and multifaceted approaches) showed conflicting results regarding cost-effectiveness [42]. While some interventions appeared cost-effective or even cost-saving, others showed no significant differences in costs or outcomes, highlighting variability in results across studies [37].

### Stratification of results

To explore the influence of methodological quality, the findings were stratified. Categorization of the determinants of guideline implementation based on theoretical frameworks was consistent across reviews, regardless of methodological quality.

Stratification showed that most implementation strategies were reported across reviews of varying methodological quality. No clear patterns were observed indicating that specific strategies were confined to higher- or lower-quality evidence. A small number of strategies were only identified in a single review, including participatory or collaborative approaches (moderate quality) [33], incentives (low quality) [28], and implementation teams and resource allocation (critically low quality) [35, 39]. However, these findings are based on limited evidence and do not indicate consistent quality-dependent patterns.

## Discussion

This umbrella review synthesized evidence from thirteen systematic reviews to evaluate the implementation of guidelines in the public healthcare sector. In line with its broad objective, the review identified a range of obstacles faced by healthcare practitioners when adapting their practice to align with guideline recommendations, as well as facilitators and strategies that can enhance adherence.

Organizational barriers emerged as the dominant influence on guideline implementation, indicating that successful uptake is largely shaped by structural conditions rather than individual behaviour alone. While organizational constraints are common across healthcare, the findings suggest they are often amplified in the public health context, which typically operates with limited resources, and less standardized infrastructure, making it harder to allocate dedicated time, staff, and facilities to guideline implementation. Unlike clinical settings with a hierarchical structure, public health systems rely on diffuse leadership and multi-sector collaboration. Because decision-making and service delivery often span multiple organizations, coordination and consistent implementation become inherently more complex.

Although less frequently discussed, factors related to the healthcare practitioner revealed that implementation is not solely dependent on knowledge or training. Rather, adoption depends on the extent to which guidelines align with existing professional roles, values, and daily practice. Public health guidelines, which are designed to prioritize collective impact and flexibility across contexts, rather than individual patient needs or professional norms, may not always translate easily into individual practitioners’ routines. Practitioners working with specific subpopulations may therefore find generalized recommendations less applicable to their daily work. Moreover, because these guidelines are intended to be implemented across diverse professional roles, individual practitioners often do not feel direct ownership of implementation. This misalignment is thereby limiting for guideline adherence.

Conversely, the findings suggest that implementation is facilitated when healthcare practitioners are actively involved in guideline development, as this enhances their perception of guidelines as relevant and applicable to practice. These insights underline the importance of designing guidelines that are not only evidence-based but also context-sensitive, user-oriented, aligned with existing workflows, and able to demonstrate visible benefits.

A broad range of implementation strategies, both single-component approaches and multi-component strategies, was identified, reflecting the complexity of addressing barriers across multiple domains. Although educational strategies were the most frequently applied single-component strategy across the reviews, their effectiveness appeared inconsistent, particularly when delivered as passive interventions. This suggests that increasing knowledge alone is insufficient to drive sustained behavioural change.

The evidence on the relative effectiveness of single-component versus multi-component strategies was mixed. Some reviews suggested no clear advantage of combining multiple strategies, while others indicated that multifaceted approaches may be more likely to produce meaningful improvements. Taken together, these findings indicate that no single strategy guarantees success. Instead, effectiveness likely depends on how well strategies are tailored to the specific context and the combination of barriers they are intended to address. Rather than focusing on specific strategies in isolation, efforts should aim to align interventions with relevant determinants at the level of the organization, the end-user, and the guideline itself. Such an approach acknowledges the dynamic interplay between these factors and may increase the likelihood of successful and sustainable implementation.

Finally, stratified analyses did not reveal clear patterns linking methodological quality to specific strategies or determinants, suggesting that key implementation themes were relatively consistent across the evidence base. However, the identification of some strategies in only a single review indicates that the evidence remains fragmented, and further high-quality research is needed to clarify which approaches are most effective under specific conditions.

In line with the observed fragmentation of the evidence, an important knowledge gap remains. The included reviews did not provide empirical evidence linking specific implementation strategies to particular barriers and facilitators. This limited the development of a practical overview that could serve as a tool to guide guideline implementation, as such an overview would necessarily rely on interpretative assumptions rather than empirically established relationships. An overview suggesting fixed relationships between determinants and strategies might therefore oversimplify the findings and imply causal links that are not sufficiently supported by the underlying evidence.

In the Netherlands, a tool has been developed, which aims to support the implementation of various innovations within the public health sector [44]. Based on scientific literature and expert consensus using the Delphi method, this tool offers a range of effective strategies per element of the four MIDI domains. While this implementation tool offers an actionable framework, its focus is broad, addressing various types of innovations across public health. In contrast, this umbrella review provides a more targeted perspective by synthesising empirical findings from international studies specifically related to the implementation of healthcare guidelines. It identifies which strategies have been applied, under what conditions, and with what outcomes, thereby validating strategic choices and informing future guideline development efforts.

### Strengths and limitations

A key strength of this umbrella review is its novelty. To our knowledge, this is the first umbrella review to specifically assess the implementation of healthcare guidelines within public health, a research area that has received relatively limited attention. By synthesising key findings published over the past decade, this review offers valuable insights to guide future guideline development.

Moreover, this review adopts a broad and integrative approach by examining both determinants of guideline implementation and strategies aimed at improving their uptake in practice. This dual focus enhances its practical relevance for researchers, policymakers, and healthcare practitioners. Additionally, based on the MIDI-tool, this review employs a unique method of data synthesis. This structured approach enabled a comprehensive thematic analysis, supporting the applicability of these findings in future implementation efforts.

From a methodological perspective, the negligible overlap of primary studies across the included reviews (CCA = 0.54%) suggests that the synthesis is unlikely to be influenced by double-counting of evidence and supports the robustness of the synthesis. In addition, the inclusion of a wide range of primary study designs, ranging from randomized trials to qualitative studies, allows for a comprehensive understanding of both effectiveness and contextual implementation factors.

However, several limitations should be considered when interpreting the findings. First, the evidence base remains relatively limited, despite the increasing emphasis on evidence-based healthcare practice. Most included reviews relied on qualitative synthesis, with only one meta-analysis available, which limits the ability to draw robust conclusions about the effectiveness of implementation strategies. Moreover, the overall methodological quality of the included reviews was variable, with the majority appraised as critically low. This raises concerns regarding the reliability and strength of the overall evidence, particularly given issues such as incomplete reporting, limited risk of bias assessment, and insufficient handling of heterogeneity.

Second, the included reviews combined evidence from both public health and clinical settings. While this reflects the available literature, it limits the extent to which findings can be attributed specifically to the public health context. Furthermore, no barriers and facilitators related to the socio-political context were identified, highlighting an important gap in the current evidence base.

Third, the synthesis was constrained by differences in the applied theoretical frameworks across reviews, such as the Theoretical Domains Framework (TDF) [26], the Capability–Opportunity–Motivation Behaviour (COM‑B) model, and the Effective Practice and Organisation of Care (EPOC) taxonomy [27, 31, 32]. Mapping these on the MIDI framework was an interpretative step, which may have influenced categorization.

## Conclusion

This umbrella review underscores that successful guideline implementation in the public health sector requires a comprehensive and systematic approach that accounts for all context-specific determinants. While most of the implementation challenges are not exclusive to public health, they are often amplified by its structural characteristics, including limited resources, diffuse governance, and multi-sector collaboration. Organizational factors emerged as key barriers, while successful implementation depends not only on knowledge but also on the alignment of guidelines with professional roles and practice contexts.

The evidence indicates that no single implementation strategy consistently leads to successful guideline uptake. Instead, effectiveness appears to depend on how well strategies are tailored to the specific context and the combination of determinants they address. This underscores the importance of considering implementation early in the guideline development process and adopting a systematic approach that integrates factors related to the organization, the end-user, and the guideline itself.

As the current evidence base within public health remains limited, rigorous, up-to-date research is required to ensure that evidence-based recommendations are effectively translated into practice and ultimately improve population health outcomes.

## Supplementary Information


Supplementary Material 1.



Supplementary Material 2.



Supplementary Material 3.


## Data Availability

All data analysed during this review is included in this published article and the supplement.

## Abbreviations

AMSTAR-2: A MeaSurement Tool to Assess Systematic Reviews
COM‑B: Capability–Opportunity–Motivation Behaviour model
EPOC: Effective Practice and Organisation of Care taxonomy
GRADE: Grading of Recommendations Assessment, Development, and Evaluation
MIDI: The Measurement Instrument for Determinants of Innovations
SMD: Standard Mean Difference
TDF: Theoretical Domains Framework
YHC: Youth Health Care
