# Supplementary material for: Implementation of healthcare guidelines in public health: an umbrella review
Source: BMC Public Health. 2026 Jun 19;26:2477. doi: 10.1186/s12889-026-28065-x (PMC13523198; doi:10.1186/s12889-026-28065-x)
Supplement: Supplementary file 2 [file 12889_2026_28065_MOESM2_ESM.docx]

Search strategies (CINAHL, Scopus, Cochrane Database of Systematic Reviews).

**Search syntax CINAHL**:

 TI (implement* OR adoption OR "quality improvement" OR disseminat* OR diffusion OR "knowledge transfer")

AND

TI ("empirically supported treatment" OR "evidence-based practice" OR "evidence-based treatment" OR "evidence-based intervention" OR guidelin*)

AND

TI (strateg* OR intervention* OR program* OR plan* OR process* OR model* OR framework* OR barrier* OR limitation* OR facilitator* OR "facilitating factor*")

AND

(PT "Systematic Review" OR PT "Meta Analysis" OR TI "systematic review" OR TI "meta-analysis")

**Search syntax Scopus**:

TITLE ( implement* OR adoption OR plan* OR "knowledge transfer" OR process* OR model* OR "quality improvement" OR disseminat* ) AND TITLE-ABS ( "empirically supported treatment" OR "evidence-based practice" OR guidelin* OR "evidence-based intervention" OR "evidence-based treatment" ) AND TITLE-ABS ( strateg* OR intervention* OR program* OR framework* OR barrier* OR limitation* OR facilitator* OR "facilitating factor*" ) AND PUBYEAR > 2013 AND PUBYEAR < 2023 AND TITLE ( "systematic review" OR "meta-analysis" ) AND ( LIMIT-TO ( LANGUAGE , "English" ) OR LIMIT-TO ( LANGUAGE , "Dutch" ) )

**Search syntax Cochrane Database of Systematic Reviews**:

(implement* OR adoption OR “quality improvement” OR disseminat* OR diffusion OR "knowledge transfer")

AND

("empirically supported treatment" OR "evidence-based practice" OR "evidence-based treatment" OR "evidence-based intervention" OR guidelin*)

AND

(strateg* OR intervention* OR program* OR plan* OR process* OR model* OR framework* OR barrier* OR limitation* OR facilitator* OR "facilitating factor*")
