# Supplementary material for: Implementation of healthcare guidelines in public health: an umbrella review
Source: BMC Public Health. 2026 Jun 19;26:2477. doi: 10.1186/s12889-026-28065-x (PMC13523198; doi:10.1186/s12889-026-28065-x)
Supplement: Supplementary file 3 [file 12889_2026_28065_MOESM3_ESM.docx]

| **Reference** | **Reason for exclusion  (full-text screening)** |
| --- | --- |
| Stokes T, Shaw EJ, Camosso-Stefinovic J, Imamura M, Kanguru L, Hussein J. Barriers and enablers to guideline implementation strategies to improve obstetric care practice in low- and middle-income countries: a systematic review of qualitative evidence. Implement Sci. 2016;11(1):144. doi:10.1186/s13012-016-0508-1 | Assessing outcomes in a specific setting (e.g. low-income) |
| Louie E, Barrett EL, Baillie A, Haber P, Morley KC. A systematic review of evidence-based practice implementation in drug and alcohol settings: applying the consolidated framework for implementation research framework. Implement Sci. 2021;16(1):22. doi:10.1186/s13012-021-01090-7 |  |
| Imamura M, Kanguru L, Penfold S, Stokes T, Camosso-Stefinovic J, Shaw B, Hussein J. A systematic review of implementation strategies to deliver guidelines on obstetric care practice in low- and middle-income countries. Int J Gynaecol Obstet. 2017;136(1):19–28. doi:10.1002/ijgo.12005 |  |
| Unverzagt S, Oemler M, Braun K, Klement A. Strategies for guideline implementation in primary care focusing on patients with cardiovascular disease: a systematic review. Fam Pract. 2014;31(3):247–266. doi:10.1093/fampra/cmt080 | Conducted strictly in a clinical setting |
| Abdullah G, Rossy D, Ploeg J, Davies B, Higuchi K, Sikora L, Stacey D. Measuring the effectiveness of mentoring as a knowledge translation intervention for implementing empirical evidence: a systematic review. Worldviews Evid Based Nurs. 2014;11(5):284–300. doi:10.1111/wvn.12060 |  |
| Yoong SL, Hall A, Stacey F, et al. Nudge strategies to improve healthcare providers’ implementation of evidence-based guidelines, policies and practices: a systematic review of trials included within Cochrane systematic reviews. Implement Sci. 2020;15(1):50. doi:10.1186/s13012-020-01011-0 |  |
| Arts DL, Voncken AG, Medlock S, Abu-Hanna A, van Weert HC. Reasons for intentional guideline non-adherence: a systematic review. Int J Med Inform. 2016;89:55–62. doi:10.1016/j.ijmedinf.2016.02.009 |  |
| Paksaite P, Crosskey J, Sula E, West C, Watson M. A systematic review using the Theoretical Domains Framework to identify barriers and facilitators to the adoption of prescribing guidelines. Int J Pharm Pract. 2021;29(1):3–11. doi:10.1111/ijpp.12654 |  |
| Forman-Hoffman VL, Middleton JC, McKeeman JL, et al. Quality improvement, implementation, and dissemination strategies to improve mental health care for children and adolescents: a systematic review. Implement Sci. 2017;12(1):93. doi:10.1186/s13012-017-0626-4 |  |
| Grady, A., Jackson, J. K., Lum, M., Delaney, T., Jones, J., Kerr, J., Falkiner, M., & Yoong, S. (2022). Barriers and facilitators to the implementation of healthy eating, physical activity and obesity prevention policies, practices or programs in family day care: A mixed method systematic review. Preventive medicine, 157, 107011. https://doi.org/10.1016/j.ypmed.2022.107011 |  |
| Sadeghi-Bazargani, H., Tabrizi, J. S., & Azami-Aghdash, S. (2014). Barriers to evidence-based medicine: a systematic review. Journal of evaluation in clinical practice, 20(6), 793–802. https://doi.org/10.1111/jep.12222 |  |
| Fønhus, M. S., Dalsbø, T. K., Johansen, M., Fretheim, A., Skirbekk, H., & Flottorp, S. A. (2018). Patient-mediated interventions to improve professional practice. The Cochrane database of systematic reviews, 9(9), CD012472. https://doi.org/10.1002/14651858.CD012472.pub2 |  |
| Pantoja, T., Grimshaw, J. M., Colomer, N., Castañon, C., & Leniz Martelli, J. (2019). Manually-generated reminders delivered on paper: effects on professional practice and patient outcomes. The Cochrane database of systematic reviews, 12(12), CD001174. https://doi.org/10.1002/14651858.CD001174.pub4 |  |
| Lau, R., Stevenson, F., Ong, B. N., Dziedzic, K., Treweek, S., Eldridge, S., Everitt, H., Kennedy, A., Qureshi, N., Rogers, A., Peacock, R., & Murray, E. (2016). Achieving change in primary care--causes of the evidence to practice gap: systematic reviews of reviews. Implementation science : IS, 11, 40. https://doi.org/10.1186/s13012-016-0396-4 |  |
| Reichenpfader, U., Carlfjord, S., & Nilsen, P. (2015). Leadership in evidence-based practice: a systematic review. Leadership in health services (Bradford, England), 28(4), 298–316. https://doi.org/10.1108/LHS-08-2014-0061 |  |
| Zamboni, K., Baker, U., Tyagi, M., Schellenberg, J., Hill, Z., & Hanson, C. (2020). How and under what circumstances do quality improvement collaboratives lead to better outcomes? A systematic review. Implementation science : IS, 15(1), 27. https://doi.org/10.1186/s13012-020-0978-z |  |
| Flodgren, G., O'Brien, M. A., Parmelli, E., & Grimshaw, J. M. (2019). Local opinion leaders: effects on professional practice and healthcare outcomes. The Cochrane database of systematic reviews, 6(6), CD000125. https://doi.org/10.1002/14651858.CD000125.pub5 |  |
| Lienhart, G., Elsa, M., Farge, P., Schott, A. M., Thivichon-Prince, B., & Chanelière, M. (2023). Factors perceived by health professionals to be barriers or facilitators to caries prevention in children: a systematic review. BMC oral health, 23(1), 767. https://doi.org/10.1186/s12903-023-03458-1 |  |
| Schipper K, Bakker M, De Wit M, Ket JC, Abma TA. Strategies for disseminating recommendations or guidelines to patients: a systematic review. Implement Sci. 2016;11(1):82. doi:10.1186/s13012-016-0447-x | Assessing patients as the target population |
| Taghizadeh, S., Hashemi, M. G., Zarnag, R. K., Fayyazishishavan, E., Gholami, M., Farhangi, M. A., & Gojani, L. J. (2023). Barriers and facilitators of childhood obesity prevention policies: A systematic review and meta-synthesis. Frontiers in pediatrics, 10, 1054133. https://doi.org/10.3389/fped.2022.1054133 (Retraction published Front Pediatr. 2024 Feb 29;12:1390512. doi: 10.3389/fped.2024.1390512. | Retracted report |
